# Is there a difference between child self-ratings and parent proxy-ratings of the quality of life of children with a diagnosis of attention-deficit hyperactivity disorder (ADHD)? A systematic review of the literature

**DOI:** 10.1007/s12402-016-0210-9

**Published:** 2016-12-22

**Authors:** Helen Galloway, Emily Newman

**Affiliations:** 1 Clinical Psychology to General Adult Psychiatry, NHS Tayside, Alloway Centre, Dundee, DD4 8UA UK; 2 Clinical and Health Psychology, School of Health in Social Science, University of Edinburgh, Edinburgh, UK

**Keywords:** Quality of life, ADHD, Attention-deficit/hyperactivity disorder, Parent–child agreement, Children, Parent

## Abstract

There are contemporary indicators that parent proxy-ratings and child self-ratings of a child’s quality of life (QoL) are not interchangeable. This review examines dual informant studies to assess parent–child agreement on the QoL of children with attention-deficit/hyperactivity disorder. A systematic search of four major databases (PsycINFO, MEDLINE, EMBASE and Cochrane databases) was completed, and related peer-reviewed journals were hand-searched. Studies which reported quantitative QoL ratings for matched parent and child dyads were screened in accordance with relevant inclusion and exclusion criteria. Key findings were extracted from thirteen relevant studies, which were rated for conformity to the recommendations of an adapted version of the STROBE statement guidelines for observational studies. In the majority of studies reviewed, children rated their QoL more highly than their parents. There was some evidence for greater agreement on the physical health domain than psychosocial domains.

## Introduction

### Quality of life in children with ADHD

Attention-deficit/hyperactivity disorder (ADHD) is, currently, the name given to describe a group of symptoms that broadly encompass inattentive, hyperactive and impulsive behaviours (with inattentive, hyperactive/impulsive and combined subtypes). There remains strong professional incongruity regarding what exactly ADHD *is*, and how it should be managed. It has consequentially received significant attention in the media. Controversies exist in relation to a number of factors, including variances in international diagnostic rates, variances in diagnostic rates within local services or between individual clinicians, anxieties about the use of stimulant medication with children, the role of pharmaceutical companies, and whether ADHD is a ‘real disorder’ or a social construct.

What exactly causes ADHD remains an unknown. It is categorised as a neuro-developmental disorder in the Diagnostic and Statistical Manual of Mental Disorders: DSM-5™ (5th ed.) (American Psychiatric Association 2013). MRI and PET scans show that changes in brain structure in the frontal regions are consistently found in children with ADHD (Krain and Castellanos 2006). However, some argue that it is not possible to assess whether brain differences are caused by (rather than being the cause of) different ways of thinking. Some also argue that stimulant medications, which are undeniably effective in reducing ADHD symptoms, would improve concentration in us all. Others are concerned that we may be unnecessarily medicalising children, and refer to ADHD as a ‘cultural construct’, where increasing rates of diagnosis are seen as a result of society’s growing intolerance to behaviour that does not conform. For a more in-depth analysis of this debate, see Timmi and Taylor (2003).

Regardless of the controversies surrounding ADHD, it remains one of the most highly prevalent health diagnoses among children and adolescents, affecting an estimated 3–7% of school-aged children (Daviss 2008), with prevalence tending to be higher among males than females (Willcutt 2012). Symptoms usually continue into adulthood and are associated with impairments in academic, social and emotional functioning (Cantwell 1996). Co-morbidity next to ADHD is the norm rather than the exception (Thompson et al. 2004) with oppositional defiant disorder (ODD), conduct disorder (CD), learning disability (LD), anxiety disorders and depression most commonly co-occurring (Biederman et al. 1991). Children who receive a diagnosis of ADHD tend to have poorer outcomes than control group children. They have an increased risk of low self-esteem, poor academic achievement, family and peer relationships problems, anti-social behaviour and criminal activity (Biederman et al. 1991). Leading neuroscientist, Dr. Bruce Perry, described the emotional dysregulation that often occurs between parents and their children when children with ADHD are struggling (Boffey 2014). He highlights the importance of implementing a combination of therapeutic approaches that aim to support parents to regulate themselves and break the cycle of negative feedback.

Available studies largely and consistently indicate that children with a diagnosis of ADHD experience impaired quality of life (QoL) (Danckaerts et al. 2010). The World Health Organisation (1995, p. 1450) defined QoL as ‘The individual’s perception of their position in life, in the context of culture and value systems in which they live, and in relation to their goals, expectations, standards and concerns’. However, until very recently, the majority of studies in this area have reported only parent proxy-measures of a child’s QoL. Therefore, the child’s subjective experience of living with ADHD remains relatively undisclosed.

In their major review of paediatric ADHD QoL studies, Danckaerts et al. (2010) reported that of the 36 studies they reviewed, 29 included parent only ratings, three studies used only child-rated measures, while only four studies utilised both parent and child ratings. The authors reported that the child self-report data were much less robust in establishing correlations between QoL and ADHD than the parent-reported data. In two of the seven studies which utilised child-reported measures, children did not consider their QoL to be more impaired than healthy controls (Klassen et al. 2006; Landgfuf and Abetz 1997). Further, some of the data from the four dual informant studies indicated that there may be some discrepancies between parent and child perceptions of the child’s QoL. One study found that children rated their QoL more positively than their parents across all domains except physical functioning (Klassen et al. 2006). Another reported discrepancies between child and parent ratings on the domains of physical health and home life (children rated higher), and bodily functions and positive moods (parents rated higher) (Flapper and Schoemaker 2008).

The review authors suggested that the child-reported data could in some way have been affected by the measures used. They highlight that the two studies where children did not rate their QoL differently from controls both used the Child Health Questionnaire (CHQ), while the four others (which used other QoL measures) reported reduced QoL. They also suggest that less robust ratings may be a result of children minimising their difficulties or an impulsive response style. Further, the authors proposed that parent ratings may be affected by the encumbrance of caring for a child with ADHD symptoms, i.e. their own QoL is affected. Indeed, some QoL studies for other conditions have reported a link between parental emotional distress and more negative perceptions of their child’s QoL (Janicke et al. 2007; Kobayashi and Kamibeppu 2011).

### Measuring paediatric quality of life

In relation to health conditions, the many available definitions of QoL emphasise the desired condition as one of general well-being, in which a person encounters a range of daily experiences, unconstrained by the potentially unpleasant and debilitating effects of a disorder. Studying QoL is particularly important in chronic conditions, where the focus of treatment is often on the management of symptoms, as opposed to being curative (Ingerski et al. 2010; Varni et al. 2007). When measuring the effectiveness of paediatric treatment interventions, there is an evolving realisation that it is not simply a reduction of symptoms that is important, but also children’s longitudinal capacity to enjoy and participate in the multi-dimensional aspects of their daily lives. Consideration must be given to whether any illness intervention can be said to be effective if it does not improve the child’s lived experience.

Generic QoL instruments are fundamentally multi-dimensional and usually contain, as a minimum, the core domains of physical, psychological, social and cognitive functioning (Eiser and Varni 2013). However, although the core domains are usually present, they are often defined differently, and instruments commonly break them down further into different sub-domains (Danckaerts et al. 2010). As a consequence, it is reasonable to assume that different QoL instruments may not always measure the same things or indeed cover the necessary ground to ascertain a full understanding of QoL. In this sense, it can be challenging to compare studies which have employed differing QoL outcome measures. Some condition-specific measures have been developed, such as the Paediatric Quality of Life Inventory (Peds-QL) cancer module (Varni et al. 2002). While these will no doubt provide detailed insight and sensitivity to the impact of a specific set of symptoms, they do not allow for comparisons with other health conditions or with normative samples.

### Parent proxy-report in paediatric QoL research

The very nature of the concept of QoL as a ‘lived experience’ should predict that the key informant would be the individual whose QoL is in question. However, studies investigating paediatric QoL have generally utilised only parent proxy-reports to provide a measure of a child’s QoL. This may be problematic, as some research has shown that parent and child reports on this concept are not interchangeable (Eiser and Morse 2001; Klassen et al. 2006). Parent proxy-measures provide, at best, an informed estimate of how a parent expects their child to feel in many (often unobserved) contexts and, at worst, a poor and misleading judgement of the internal world of a child into whom they have little, or misconstrued, insight. This pattern has in the past been justified by the belief that children had not yet achieved a sufficient level of cognitive and linguistic development to enable self-completion of QoL measures (Upton et al. 2008). However, several instruments designed to measure self-rated QoL in children as young as five have been developed in recent years [e.g. Paediatric Quality of Life Inventory (Peds-QL)] (Varni et al. 1999); the CHQ (Landgraf et al. 1996); and KIDSCREEN (Ravens-Sieberer et al. 2007), and research has demonstrated that children are able to reliably assess their own QoL (Cremeens et al. 2006; Varni et al. 2007).

The use of child-rated measures does not render parent perceptions redundant, however. When a child is too impaired to express her views, or is unwilling, parent ratings may be the only available option. Additionally, parent accessing of healthcare and support services for their child is, in the main, predicted by their perceptions of their child’s QoL (Varni et al. 2001). Further, the level of concordance between parents and their children in assessing the child’s QoL could potentially have significant clinical relevance to chronic conditions. A comparison of both perspectives could offer clinicians valuable insight into how features of the condition might affect the child’s internal perceptions relative to others’ external perceptions. Simply put, there may be no ‘true representation’ of the child’s QoL, rather than both perspectives are likely to relay important information regarding the nature and impact of the condition. Assessing both perspectives may also provide insight into the nature of the relationship between parent and child and the expectations they individually possess regarding the condition and available treatments. Investigating the sources of any discrepancies which arise between them may in turn influence clinical decision-making regarding key areas for intervention.

### Parent–child concordance on QoL measures

Previous reviews have investigated parent–child agreement on QoL measures, featuring study samples of children with a range of chronic health diagnoses and healthy control groups (Eiser and Morse 2001; Eiser and Varni 2013; Upton et al. 2008). These reviews report consistent discrepancies between parent proxy-reports and child self-reports. It is possible that these discrepancies reflect a wider perceptual issue between self and proxy-raters in general. However, studies have suggested that parents of healthy children generally rate them as having better QoL than the children rate themselves (Jozefiak et al. 2008), while this trend is reversed in children with health conditions (Eiser and Morse 2001; Upton et al. 2008). This would suggest there is a relationship between the child’s health status and how children and their parents perceive the child’s experiences.

Inter-rater agreement is often highest for objective, externalising domains like walking, running, aggression, school refusal and hyperactive behaviour, while there is generally less concordance for internalising, emotion-based domains such as fatigue, pain, sadness and worry (Eiser and Morse 2001). This suggests that parents are better at interpreting their child’s observable behaviour than their internal state of mind. However, this trend can be found to be reversed in the literature both within and between different health conditions (e.g. van Gent et al. 2007; Czyzewski et al. 1994).

The findings described offer valuable insights into patterns of concordance between child and parent reports across QoL studies for children with a range of health conditions. However, comparing samples across conditions can be problematic given that definitions of a diagnosis can be broad (e.g. cancer) and that each condition will have its own symptoms, treatments and prognosis. Thus, the individual domains of QoL measures may be affected, to a greater or lesser degree, by each condition. Potentially, this will lead to differing levels and patterns of concordance between children and their parents on QoL measures. This issue highlights a need for condition-specific research which utilises parent and child ratings of the child’s QoL, so that the unique contributing factors of the associated symptomatology can be explored.

Due to the small number of studies incorporating children’s views, any existing inconsistencies between parent and child perceptions of child QoL are not well studied in the context of ADHD. A focussed review of further studies is necessary to explore the patterns of concordance between child and parent perceptions in detail and to deduce what factors might underlie any discrepancies. Since Danckaerts et al.’s (2010) review, a number of additional studies have been published which have reported both child- and parent-rated measures of child QoL. A systematic review of this material is now warranted.

## Aim of the review

The aim of this review was to systematically examine the existing empirical data regarding the level of agreement between parent proxy- and child self-report ratings of the QoL of children with a diagnosis of ADHD, as measured by quantitative QoL instruments.

## Method

### Inclusion and exclusion criteria

#### Population

Studies were included where the target population was children with a diagnosis of ADHD aged 0–18 years. Samples were included regardless of whether co-morbidities were present or had been purposely excluded.

#### Study design

In the light of the nature of the research question, it was anticipated that observational studies would be most prevalent, of cross-sectional, case–control and cohort study design. However, randomised controlled trials (RCTs) were not excluded from the review. Studies were included if they used a quantitative design and either compared or reported data (sample size, means and standard deviations) from QoL measures for matched parent and child dyads. Where treatment outcome studies were included, baseline QoL measures were used. Studies which provided only child self-reports or parent/carer proxy-reports were excluded. Studies where someone other than the parent/carer was the proxy-rater (e.g. teachers or clinicians) were excluded. Studies which utilised control groups or a single group sample were included. Due to issues of generalisability and increased bias, single case studies were excluded.

#### Outcome measures

Studies were included if they aimed to measure the QoL of children with a diagnosis of ADHD, using a standardised QoL instrument with established psychometric properties. To enable a meaningful inter-rater comparison of QoL data, only studies which featured instruments that measured the same content and constructs for self and proxy-reports, using parallel questions and rating scales, were included. QoL measures which utilised a single item measure were not included.

#### Language

Studies that were not published in the English language were excluded from the review due to a lack of translation resources available to the reviewer.

### Literature search strategy and study selection

**Fig. 1:**
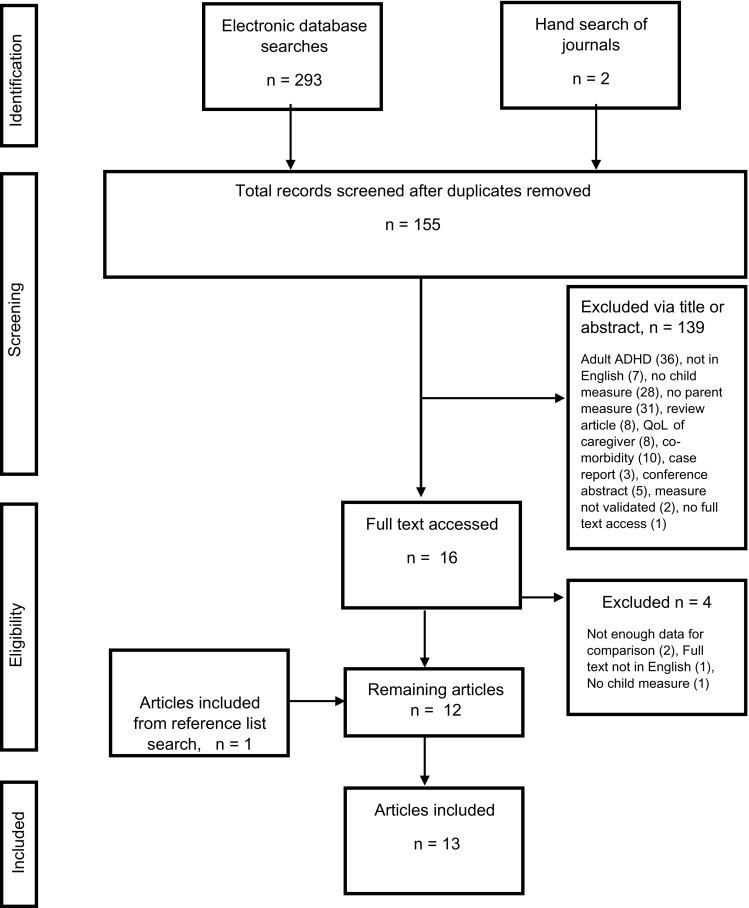
Flow chart detailing the study selection and exclusion process

Study selection was achieved by completing literature searches of electronic databases and hand searching of specific relevant electronic journals. Reference lists from studies selected for inclusion were also reviewed (see Fig. 1).

#### Electronic database searches

The databases searched were PsycINFO (1806–January 2015), EMBASE (1974–January 2015), MEDLINE (1946–January 2015) and Cochrane Library database (1999–January 2015). The databases were searched by entering the terms (*ADHD* OR *attention deficit*/*hyperactivity disorder*) AND (*QoL*) within the domains of title, abstract and keyword/subject heading. A total of 153 items were returned using this search strategy after duplicates were removed.

#### Hand searching of selected journals

Three journals were hand-searched based on their relevance to the research area or their frequency as publishers of the studies that met the inclusion criteria from the database searches. These were: ADHD: attention deficit and hyperactivity disorders; European Journal of Child & Adolescent Psychiatry; and Journal of Attention Disorders. These journals were searched from the year 2004–2015 (January). This search returned two potentially relevant studies for further screening.

#### Reference list searches

One further study was obtained using the snowball technique (i.e. reviewing the reference lists of studies which met the inclusion criteria) (Schei et al. 2013).

### Study appraisal process

Assessing the quality of research studies and their partiality is paramount when conducting systematic reviews and meta-analyses and interpreting results. Formal quality assessment tools are increasingly well developed in the context of clinical trials and RCTs (Deeks et al. 2003). However, less consideration has been given to the use of similar instruments for appraising observational studies. Recent reviews have concluded that there is no one distinct tool advocated for this task (Jarde et al. 2012; Sanderson et al. 2007). For the current review, the STROBE (von Elm et al. 2007) statement guidelines for observational studies have been utilised to evaluate the quality of the included studies. Although the intended purpose of STROBE was to act as a reporting guide to authors of observational studies, it has been endorsed by researchers as a starting point for the methodological appraisal of non-experimental studies (Sanderson et al. 2007). Its popularity may be attributed to the comprehensive method of its development and the presence of criteria that appear to evaluate bias (Sanderson et al. 2007).

The quality review does not provide a comparative measure across included studies, given that each of the recommendations is not equally weighted. It does, however, provide an indication as to whether the recommended methodological and reporting aspects of the research process were present for each study. Issues relating to research methodology allow readers to assess how well a study has been designed and conducted and therefore consider how valid and generalisable the results can be assumed to be. Issues relating to reporting of research allow readers to consider how well authors have detailed, explained and/or interpreted their methods and findings. For the purpose of this review, methodological conformity to the recommendations took precedence over reporting conformity, given that it is the results of each study, rather than their interpretation by the authors, which are most relevant to the research question.

Some adaptations were made to the criteria in order to increase their relevance to the research question. The main adaptations reduced the number of unnecessary criteria or reworded given criteria to reflect methodological quality rather than reporting quality. The adaptations are presented in Table 1. Conformity to the items of the adapted STROBE statement guidelines was rated for each of the included studies using a binary judgement (Yes/No). A further rating of N/A (not applicable) was applied where appropriate. The recommendations comprise of six main areas (Title/Abstract, Introduction, Methods, Results, Discussion and Other Information), some of which incorporate sub-items (for the full guidelines, see ‘Appendix’). A comprehensive definition of each recommendation is detailed in Vandenbroucke (2007). All thirteen papers were independently coded by the first author, and a randomly generated sample of six papers (46.2%) was cross-rated by the second author. The inter-rater reliability was found to be 0.79 (*p* < .001), indicating ‘substantial’ reliability (Landis and Koch 1977).

**Table 1:** Adaptations to the STROBE checklist criteria

Criterion 1	Title/abstract—reduced to one criterion
Criterion 4	Setting—broken down to further criteria of (a) ‘location’ and (b) ‘relevant dates’
Criterion 8	Measurement—altered to indicate use of valid/reliable outcome measures appropriate to the population and for use with parent/child dyads
Criterion 9	Bias—altered to indicate active control for bias rather than the authors’ description
Criterion 12	Statistical methods: parts (b), (d) and (e) removed
Criterion 13	Participants—part (c) ‘consider use of a flow diagram’ removed
Criterion 16	Main results—reduced to one criterion
Criterion 19	Limitations—broken down to further criteria of (a) ‘sources of potential bias or imprecision’ and (b) ‘direction and magnitude of potential bias’

## Results

Thirteen studies that met the inclusion criteria were identified (Table 2). The studies were published across nine different countries: USA (3); the Netherlands (2); Norway (2); Iran (1); Thailand (1); Canada (1); Australia (1); Brazil (1); and Turkey (1).

**Table 2:** Characteristics of included studies

Author (year), country	QoL Measure	Sample characteristics (all ADHD samples include matched QoL dyads)	Design	Main findings relevant to current review (*p* values shown where reported)	Database	Co-morbidities	Domains
Thaulow and Jozefiak (2012), Norway	ILC	Age range 8–15.5 62 ADHD groups, 23 girls (37.1%), 39 boys (62.9%) 49 anxiety/depression groups, 20 girls (40.8%), 29 boys (59.2%) 65 healthy control groups, 25 girls (38.5%), 40 boys (61.5%)	Case–control (comparison of QoL in children with ADHD and children with anxiety/depression)	In the ADHD group, children’s self-reported QoL was significantly higher than the parent-reported QoL (*p* < .01). The same analysis did not find a significant difference for the anxiety/depression group. Children in ADHD group rated QoL as being higher than anxiety/depression group (*p* < .05), lower than healthy controls (*p* < .05)	N/A	Not excluded	No only totals for QoL measure.
Sciberras et al. (2011), Australia	Peds-QL	Age range 8–18 47 ADHD children 45 boys, 2 girls Aged 8–12: (36) Aged 13 or over: (11)	Cross-sectional (children’s experiences of ADHD)	Children rated their QoL higher than their parents rated them for total QoL scores and on all domains except physical functioning. Total: mean diff. = −11.6, 95% CI’s −18.64 to −4.70, *p* < .001. Psychosocial: mean diff. = −14.64, 95% CI’s −22.04 to −7.24, *p* < .01. Emotional: mean diff −14.15, 95% CI’s −23.56 to −4.74, *p* < .001. Largest mean differences were in social: mean diff.: −16.49, 95% CI’s −26.41 to −6.57, *p* < .001; and School: mean diff −15.11, 95% CI’s −23.01 to −7.21, *p* < .001 domains	PsycINFO	Not excluded	Yes
Marques et al. (2013), Brazil	Peds-QL	Age range 8–12 45 ADHD 43 control (age and gender not specified)	Cross-sectional (with comparison group) (parent–child comparison of QoL)	Both children and parents rated QoL as lower in ADHD group than control group on all domains. Good concordance between parents and children on all domains except school functioning, which children self-reported higher than parents (mean difference: 14.55, CI 95%: 7.77, 21.33, SD: 3.36).	MEDLINE	Specified no co-morbidities in ADHD group except ODD	Yes
Limbers et al. (2011a), USA	Peds-QL	Age range 5–18 179 children (57 girls, 124 boys) 181 parents (177 matched dyads)	Cross-sectional (paediatric clinic vs mental health clinic)	Children rated QoL higher than parents for total QoL score (mean diff. = 8.57, *p* < .001, *d* = 0.5). Children rated QoL higher across all domains except physical health. Greatest discrepancies were on school functioning (mean diff. = 12.06, *p* < .001, *d* = 0.61), and psychosocial health (mean diff. = 10.18, *p* < .001, *d* = 0.57)	EMBASE	Not excluded	Yes
Limbers et al. (2011b), USA	Peds-QL	Age range 5–18 ADHD group 1: 17 general paediatric clinic (10 boys, 7 girl) and parents. ADHD group 2: [see Limbers et al. (2011a)]. Healthy controls from previous sample (957 boys, 496 girls)	Case–control (validation of Peds-QL in a sample of children with ADHD and co-morbid psychiatric disorders)	Group 1: no statistically significant differences between parent- and child-rated mean QoL scores^a^, but sample size was small (*N* = 17) Group 2: [see Limbers et al. (2011a)]	PsycINFO	Not excluded	Yes
Flapper and Schoemaker (2008), The Netherlands	DUX-25 and TACQOL	Age range 7–10 years 8 months ADHD + DCD (developmental coordination disorder) 23, control 23	Double-blind placebo-controlled clinical trial (effects of methylphenidate on QoL)	DUX-25: no significant differences between parent and child reports for total scores but over some domains (physical *p* < .001, home *p* < .004) (children self-rated higher) TACQOL: bodily functioning (*p* < .02) and positive moods (*p* < .02) (parents rated higher) (baseline scores analysed)	PsycINFO	Excluded except for developmental coordination disorder (DCD)	Yes
Varni and Burwinkle (2006), USA	Peds-QL	Age range 5–16 72 ADHD dyads 60 boys (83.3%), 12 girls (16.7%) 66 cancer, 57 cerebral palsy, 3256 healthy controls	Case–control (population based Peds-QL validation study)	Good reliability for total scale score (Cronbach’s *α* .92 child self-report, .92 parent proxy-report) and across domains Effect sizes (parent, child): total, .71, .70, physical .67, .67, psychosocial, .69, .69, emotional .67, .66, social .75, .75, school, .59, .59	MEDLINE	Excluded	Yes
Klassen et al. (2006), Canada	CHQ	Age range 10–17 58 dyads Male 48 (82.8%), female 10 (17.2%)	Cross-sectional (parent and child comparison on QoL)	Children self-reported significantly higher QoL than parents on 4 domains: behaviour (mean diff. = 22.9, 95% CI’s 17.6–28.3, SD 19.8, *p* < .001), self-esteem (mean diff. = 14.7, 95% CI’s 8.2–21.1, SD 23.7, *p* < .001), mental health (mean diff. = 6.8, 95% CI’s 1.6–12.0, SD 19.2, *p* < .01), family cohesion (mean diff. = 10.6, 95% CI’s 3.7–17.5, SD 25.7, *p* < .01), and worse on one: physical function (mean diff. = −4.3, 95% CI’s −7.8 to −0.8, SD 13.2, *p* < .01) Discrepancies were related to co-morbid ODD, worse ADHD symptoms and psychosocial stressors	PsycINFO	Excluded	Yes
Schei et al. (2013), Norway	ILC	Age range 13–18 194 dyads Male 55.2%	Case–control (impact of emotional and conduct problems on QoL in ADHD)	For the ADHD only group, adolescents reported higher total QoL scores than parents (*p* < .01). There were no significant differences between total QoL scores for parents and adolescents for the ADHD with additional emotional problems group or the ADHD with additional conduct problems group. No subdomain scores were reported, only totals	N/A	Excluded for direct comparison with ADHD + co-morbid conditions	No only totals
Jafari et al. (2011), Iran	Peds-QL	Age range 8–17 72 dyads, 140 controls 8–12 year (75%), 13–18 years (25%)	Cross-sectional (parent and child comparison on QoL)	Children rated their total QoL as higher than parents (mean diff. = 5.33, 95% CI’s −10.6 to −0.06, *p* < .05)^a^. They also rated higher mean QoL scores for the school domain (mean diff. = 8.9, 95% CI’s −16.62 to −1.2, *p* < .05)^a^ Parents and children in ADHD group rated QoL significantly poorer than control group across all domains	PsycINFO	Information not provided	Yes
Pongwilairat et al. (2005), Thailand	Peds-QL	46 ADHD 94 healthy control children (information not provided)	Cross-sectional (QoL of children with ADHD)	Means show children rate their total QoL higher than parents (mean diff. = 146, 95% CI’s 20.1–272.2, *p* < .05)^a^. Children also rated QoL higher on physical domain (mean diff. = 84.24, CI’s 22.2–146.3, *p* < .01)^a^; however, differences on other subdomains were not significant. Children and parents rated QoL poorer than healthy controls, except children self-report no significant difference in physical health domain, while parents do	EMBASE	Information not provided	Yes
Bastiaansen et al. (2004), The Netherlands	Peds-QL	Age range 6–18 310 dyads 107 ADHD 57 anxiety 28 developmental disorders 29 mood disorders 22 other disorders 67 no diagnosis	Cross-sectional (QoL in children with psychiatric disorders)	For ADHD group, children self-reported higher mean QoL scores than parents across all domains Total: mean diff. = 6.6, 95% CI’s 3.1–10.1, *p* < .001^a^ Psychosocial: mean diff. = 8, 95% CI’s 3.9–12.1, *p* < .001^a^ Physical: mean diff. = 4, 95% CI’s 0.02–8.0, *p* < .05^a^ Emotional: mean diff. = 6.4, 95% CI’s 1.25–11.5, *p* < .05^a^ Social: mean diff. = 11.2, 95% CI’s 7.1–15.3, *p* < .001^a^ School: mean diff. = 6.6, 95% CI’s 2.01–11.9, *p* < .05^a^	PsycINFO	Excluded for direct comparisons with other conditions	Yes
Gürkan et al. (2010)	Peds-QL	Age range 8–14 45 dyads (75.6%) 34 boys (24.4%) 11 girls	Open-label trial (psychiatric symptoms and methylphenidate)	Children rated their QoL better than their parents for total QoL score at baseline (mean diff. = 5.4, 95% CI’s 0.2–10.6, *p* < .05)^a^. No significant differences observed for psychosocial or physical domains	EMBASE	Excluded	Yes

*QoL* quality of life, *Peds*-*QL* Paediatric Quality of Life Inventory 4.0 generic core scales (Varni et al. 1999), *ILC* inventory of life quality in children and adolescents (Mattejat and Remschmidt 2006), *CHQ* Child Health Questionnaire (Landgraf et al. 1996), *DUX*-*25* Dutch-Child-AZL-TNO-Quality-of-Life (Kolsteren et al. 2001), *TACQOL* TNO-AZL-Child-Quality-of-Life (Vogels et al. 1998)

^a^Statistical analysis of mean differences between groups was carried out by the author of the review based on data reported in the article

### Characteristics of included studies

Table 2 lists the included studies and provides an overview of the main findings of each study as relevant to the research question. Seven were cross-sectional in design, four were case–control studies, one was an open-label trial, and one was a double-blind, placebo-controlled clinical trial.

### Sample characteristics

In total, 13 studies included 967 matched parent–child dyads, where the child had a diagnosis of ADHD. The number of dyads in each study ranged from 17 to 194. Children ranged in age from 5 to 18 years. In general, boys represented a higher proportion of the samples, ranging from 55.2 to 95.7%.

Four of the studies in the review excluded participants with conditions co-occurring with ADHD (Bastiaansen et al. 2004; Flapper and Schoemaker 2008; Schei et al. 2013; Varni and Burwinkle 2006), six did not exclude participants with co-morbidities at all (Klassen et al. 2006; Thaulow and Jozefiak 2012; Sciberras et al. 2011; Limbers et al. 2011a, b; Gürkan et al. 2010), one study limited co-morbidities to ODD (Marques et al. 2013), and two studies did not provide information about whether or not co-morbidities were excluded (Jafari et al. 2011; Pongwilairat et al. 2005).

### Quality of life measures

Five unique QoL measures were utilised by the studies included in the review: the Paediatric Quality of Life Inventory (Peds-QL) (Varni et al. 1999); the Inventory of Life Quality in Children and Adolescents (ILC) (Mattejat and Remschmidt 2006); the CHQ (Landgraf et al. 1996); the Dutch-Child-AZL-TNO-Quality-of-Life (DUX-25) (Kolsteren et al. 2001); and the TNO-AZL-Child-Quality-of-Life (TACQOL) (Vogels et al. 1998). All of these instruments have been demonstrated to have acceptable psychometric properties. Nine (69.2%) of the studies used the Peds-QL (Bastiaansen et al. 2004; Gürkan et al. 2010; Jafari et al. 2011; Limbers et al. 2011a, b; Marques et al. 2013; Pongwilairat et al. 2005; Sciberras et al. 2011), 2 (15.4%) used the ILC (Schei et al. 2013; Thaulow and Jozefiak 2012), 1 (7.7%) used the CHQ (Klassen et al. 2006), and 1 (7.7%) used both the DUX-25 and the TACQOL (Flapper and Schoemaker 2008). Despite the popularity of the Child Health Illness Profile (CHIP) in ADHD studies, the authors did not find any studies which utilised this measure in a way that met criteria for inclusion in this review.

### Statistical analyses

A range of different statistical analyses were utilised among the included studies in order to compare parent and child ratings. Six studies used *t* tests (Thaulow and Jozefiak 2012; Sciberras et al. 2011; Limbers et al. 2011a; Flapper and Schoemaker 2008; Klassen et al. 2006; Schei et al. 2013), and one study used the Bland–Altman method (Marques et al. 2013). Three studies utilised Cronbach’s alpha coefficients and Pearson intra-class correlations to compare levels of concordance between parent- and child-rated measures (Bastiaansen et al. 2004; Klassen et al. 2006; Varni and Burwinkle 2006).

For five studies, it was necessary for the author to carry out additional statistical analysis to directly compare the QoL data reported for the purpose of the review (Jafari et al. 2011; Pongwilairat et al. 2005; Limbers et al. 2011b; Gürkan et al. 2010; Bastiaansen et al. 2004). These studies all reported the number of participants in each comparison group, means for the total and domain QoL scores for parents and children, as well as the relevant standard deviations. With this information, the author was able to estimate whether there were statistically significant differences between the two groups of data using an online *t* test calculator, GraphPad data analysis software (http://www.graphpad.com/quickcalcs/ttest1/?Format=SD).

### Quality ratings of included studies

 Table 3 presents an overview of how closely the thirteen reviewed studies’ conformed to the recommendations from the adapted STROBE guidance statement.

**Table 3:** Quality ratings of included studies

Items (Y/N)	Thaulow and Jozefiak (2012)	Sciberras et al. (2011)	Marques et al. (2013)	Limbers et al. (2011a)	Limbers et al. (2011b)	Flapper and Schoemaker (2008)	Varni and Burwinkle (2006)	Klassen et al. (2006)	Schei et al. (2013)	Jafari et al. (2011)	Pongwilairat et al. (2005)	Bastiaansen et al. (2004)	Gürkan et al. (2010)
1. Title and abstract	Y	Y	Y	Y	Y	Y	Y	Y	Y	Y	Y	Y	Y
2. Rationale	N	Y	Y	Y	Y	Y	Y	Y	Y	Y	N	Y	Y
3. Objectives	N	Y	Y	Y	Y	Y	Y	Y	N	Y	N	Y	Y
4. Study design	Y	Y	Y	Y	Y	Y	Y	N	Y	Y	N	Y	Y
3. (a) Location	Y	Y	Y	Y	Y	Y	Y	Y	Y	N	Y	Y	Y
(b) Relevant dates	Y	Y	N	Y	N	N	Y	Y	Y	N	N	Y	N
6. (a) Participants	N	Y	Y	Y	Y	Y	Y	Y	Y	N	N	Y	Y
(b) Control group	Y	N/A	N	Y	N	N	Y	N/A	Y	Y	N	N/A	N/A
7. Variables	Y	N	Y	Y	Y	Y	Y	Y	Y	N	Y	Y	Y
8. Measurement	Y	Y	Y	Y	Y	Y	Y	Y	Y	Y	Y	Y	Y
9. Bias	Y	Y	N	Y	Y	Y	Y	N	Y	N	N	N	Y
10. Study size (rationale)	N	N	N	N	N	Y	N	N	N	N	N	N	N
11. Quantitative variables	Y	Y	Y	Y	Y	Y	Y	Y	Y	N	Y	Y	Y
12. (a) Statistical methods	Y	Y	Y	Y	Y	Y	Y	Y	Y	Y	Y	Y	Y
(c) Missing data	Y	N	N	N	N	N	Y	N	Y	N	Y	N	N
13. (a) Participants	Y	Y	Y	N	Y	Y	Y	Y	Y	N	Y	Y	Y
(b) Non-participation	Y	N	N	N	N	Y	N	Y	Y	N	N	N	Y
14. (a) Sample characteristics	Y	Y	N	Y	Y	Y	Y	Y	Y	N	Y	Y	Y
(b) Missing data	N	N	N	Y	N	N	Y	N	Y	N	Y	Y	N
15. Outcome data	Y	Y	Y	Y	Y	Y	Y	Y	Y	Y	Y	Y	Y
16. Main results	Y	Y	Y	Y	Y	Y	Y	Y	Y	Y	Y	Y	Y
17. Other analyses	Y	Y	Y	Y	N/A	Y	Y	Y	N/A	N/A	N/A	Y	Y
18. Key results	Y	Y	Y	Y	Y	Y	Y	Y	Y	Y	Y	Y	Y
19. Limitations (a) sources	N	Y	Y	Y	Y	N	Y	Y	Y	N	Y	Y	N
(b) Magnitude	N	N	N	N	N	N	Y	Y	N	N	N	N	N
20. Interpretation	Y	Y	N	Y	Y	Y	Y	Y	Y	Y	N	Y	Y
21. Generalisability	Y	Y	N	Y	Y	N	Y	Y	Y	Y	N	Y	N
22. Funding	Y	Y	Y	Y	Y	N	Y	N	Y	N	N	Y	Y

The included studies varied significantly in terms of their conformity to the applied quality criteria. Two of the papers in particular did not meet a large number of the criteria (Jafari et al. 2011; Pongwilairat et al. 2005). There is some doubt therefore, as to whether these studies in particular applied the necessary methodological rigour to achieve a valid or representative result. They are included in the review; however, results are discussed with their methodological issues in mind.

In addition, across the range of studies, there were a number of criteria which authors commonly failed to report or address, as exemplified in the STROBE elaboration paper (Vandenbroucke 2007). The most commonly unreported methodological issues were not providing a rationale for how study size was calculated (item 10; 12 studies did not address), not addressing how missing data were handled in the statistical analysis of results (item 12c; nine studies did not address), not giving reasons for non-participation (item 13b; eight studies did not address), and not providing the number of participants with missing data at each stage of the study (item 14b; eight studies did not report). The most commonly unaddressed reporting issues were: not reporting the relevant dates/time period within which data were collected (item 5b; six studies did not report) and failing to discuss the direction and magnitude of the limitations reported (item 19b; 11 studies did not report). These issues, although important, are less likely to directly impact results. Given these issues, the findings presented in this review should therefore be interpreted cautiously.

### Summary of results

#### Parent–child agreement on total QoL scores

Total QoL scores were available for twelve of the thirteen included studies. Eight of the twelve studies (66.6%) reported significantly higher child self-reported total QoL scores when compared with parent proxy-reported QoL scores (Sciberras et al. 2011; Limbers et al. 2011a; Jafari et al. 2011; Pongwilairat et al. 2005; Bastiaansen et al. 2004; Gürkan et al. 2010; Thaulow and Jozefiak 2012; Schei et al. 2013). Four of the studies (33.3%) reported no statistically significant differences in total QoL scores (Marques et al. 2013; Varni and Burwinkle 2006; Limbers et al. 2011b; Flapper and Schoemaker 2008). One study did not report a total QoL score, only domains (Klassen et al. 2006).

#### Parent–child agreement across outcome measures

In six of the nine studies which utilised the Peds-QL (66.6%), children rated their overall QoL significantly higher than their parents rated their QoL (Sciberras et al. 2011; Limbers et al. 2011a; Jafari et al. 2011; Pongwilairat et al. 2005; Bastiaansen et al. 2004; Gürkan et al. 2010). In the three remaining studies which used the Peds-QL, no significant differences were found between overall parent and child ratings of QoL (Marques et al. 2013; Varni and Burwinkle 2006; Limbers et al. 2011a). One of these three studies utilised a relatively small sample size compared to the others in the review (*n* = 17) (Limbers 2011). Both of the two studies which utilised the ILC reported that children rated their overall QoL significantly higher than their parents rated them (Schei et al. 2013; Thaulow and Jozefiak 2012). The study which utilised the TACQOL and the DUX-25 reported no significant differences in total QoL scores (Flapper and Schoemaker 2008). The study which utilised the CHQ did not report total QoL scores but did report significant discrepancies across domains in the direction of children rating QoL higher than their parents (Klassen et al. 2006).

#### Parent–child agreement on QoL domains

Across the whole sample of studies, 11 (84.6%) reported data for QoL domains. Individual domain scores were not reported by either of the ILC studies (Schei et al. 2013; Thaulow and Jozefiak 2012). One study reported discrepancies across all domains (Bastiaansen et al. 2004). Four of the eleven studies (36.4%), all using the Peds-QL, reported higher parent–child agreement on physical health than on psychosocial domains (social, school and emotional experience) (Sciberras et al. 2011; Marques et al. 2013; Limbers et al. 2011a; Jafari et al. 2011). In one study, also using the Peds-QL, this trend was reversed, with greater agreement on psychosocial domains than physical domains (Pongwilairat et al. 2005).

Klassen et al. (2006) using the CHQ reported that the direction of the observed discrepancies between child and parent ratings was different for physical (children rated lower) versus psychosocial domains (children rated higher), suggesting significant directional differences in parent and child perceptions according to the type of domain in question. Flapper and Schoemaker (2008) reported discrepancies across both observable and subjective domains. Children rated themselves as having better QoL in the ‘physical’ and ‘home’ domains (using the DUX-25), while they rated poorer QoL on ‘bodily functioning’ and ‘positive moods’ domains than the parent-rated QoL (using the TACQOL). Varni and Burwinkle (2006); Limbers et al. (2011b); and Gürkan et al. (2010) reported no discrepancies between parents and children across all individual domains.

#### Parent–child agreement in comparison to normative QoL data

Nine of the thirteen studies compared data from the ADHD group with normative data; however, one of these did not compare total QoL scores, only domain scores (Klassen et al. 2006). In all of the eight studies which did compare total QoL scores, both parents and children rated the overall QoL of the child with ADHD as poorer than the QoL of a designated healthy control group (Thaulow and Jozefiak 2012; Marques et al. 2013; Limbers et al. 2011a, b; Varni and Burwinkle 2006; Jafari et al. 2011; Pongwilairat et al. 2005; Flapper and Schoemaker 2008). There were some exceptions to this on individual domains. Limbers et al. (2011b) found that children did not rate their QoL as being significantly different from controls on the ‘social’ domain, while parents did not rate their children’s physical health as being significantly poorer. Similarly, Varni and Burwinkle (2006) reported that parents did not rate their child as having impaired physical health, while Pongwilairat et al. (2005) reported that children did not perceive their physical health as comparatively lacking. Klassen et al. (2006) found that children self-rated their QoL similarly to a normative sample across most domains, while their parents perceived deficits in psychosocial and family domains.

#### Direction of differences

Overwhelmingly, the directional trend across the range of included studies was that children reported better QoL than their parents’ proxy-ratings of QoL. All eight of the studies where there was significant parent/child discrepancies in total QoL reported higher scores for self-rated QoL. The vast majority of discrepancies across individual domains followed the same directional trend as the overall scores. Children rated higher self-rated QoL than parent-rated QoL on nineteen individual domains across eight studies. Parents rated higher QoL than children on only three individual domains across two studies. These were ‘positive moods’ and ‘bodily functioning’ using the TACQOL (Flapper and Schoemaker 2008) and the ‘physical’ domain using the CHQ (Klassen et al. 2006).

#### Co-morbidities

Two of the four studies which excluded participants with co-morbid conditions (ADHD only) found that children rated their QoL higher than their parents (Bastiaansen et al. 2004; Schei et al. 2013). Both of these studies utilised comparison groups of children with either ADHD and other conditions (e.g. emotional/CDs) (Schei et al. 2013) or other conditions without ADHD (Bastiaansen et al. 2004). Parent/child disagreement was not observed for either of these conditions as it had been in the ADHD only conditions. Four of the six studies which did not exclude co-morbid conditions reported that children rated their total QoL as being higher than their parents rated them (Thaulow and Jozefiak 2012; Limbers et al. 2011b; Sciberras et al. 2011; Gürkan et al. 2010). The fifth study (Klassen et al. 2006) did not report overall QoL scores, but did report that children with ODD/CD were more likely to rate their QoL on ‘mental health’ and ‘behaviour’ domains more favourably than their parents. Only one of the studies where co-morbidities were present found no significant differences between self- and parent-reported QoL (Limbers et al. 2011b). The study which limited co-morbidities to ODD reported good concordance between parents and children for overall QoL ratings (Marques et al. 2013). Both of the studies which did not provide information about whether or not co-morbidities were excluded reported that child ratings of QoL were higher than parent ratings (Jafari et al. 2011; Pongwilairat et al. 2005).

#### Parent and child characteristics

The relationship of the parent to the child (mother, father or legal guardian) may affect inter-rater agreement, but most studies did not deviate from ‘parent’ as the solo descriptor of the proxy-rater. Information regarding associated variables such as parent mental and physical health status, the child’s age and gender would also have been potentially valuable. However, few of the papers reported the impact of these variables on parent/child agreement in their analysis (some citing small sample sizes), so meaningful comparisons were not possible. Medication status was also of interest in this review; however, its purpose was not to assess QoL of children with ADHD according to medication status or type. As most children were recruited from paediatric clinics, the rates of pharmacological intervention were high, and few studies exclusively compared parent and child QoL ratings between medicated and non-medicated children.

## Discussion

Reviews comparing child self-report with parent proxy-reports of the QoL of children with chronic health conditions have found that inter-rater discrepancies are common and that one cannot simply be substituted for the other (Eiser and Morse 2001; Upton et al. 2008). The aim of this review was to examine the existing published data on QoL in childhood ADHD, as rated by matched parent/child dyads, in order to determine the degree and nature of any differences which occurred between them.

### Agreement on total quality of life scores

In the majority of studies, there was disagreement between parents and children on the evaluation of the child’s life quality. In all of the studies where discrepancies in overall QoL scores existed, children perceived their QoL as being more favourable than parents. These findings indicate that children with ADHD have a more positive view of their lives than their parents expect them to. These findings are in accordance with previous reviews which found that children with chronic conditions tend to rate their QoL more highly than their parents (Eiser and Morse 2001; Upton et al. 2008). In the majority of cases, both parents and children agreed that QoL is impaired for children with ADHD compared with healthy children. This evidence expands on Danckaerts et al.’s (2010) finding that QoL is impaired in children with ADHD according to parental report. Therefore, rather than informants disagreeing on whether or not impairment in QoL exists for children with ADHD, it appears that it is the level and/or the nature of the impairment on which there are often perceptual differences.

Several explanations have been offered for the propensity for children with ADHD to self-rate their QoL more favourably than their parents rate them. A *positive illusory bias*, which proposes that children with ADHD have overly optimistic self-perceptions, has been reported in studies exploring self-concept in ADHD (Hoza et al. 2002; Owens and Hoza 2003). It has been hypothesised that children with this diagnosis cope with negative experiences and protect their self-image by constructing a more favourable internal representation of their competences (Ohan and Johnston 2010). Clearly if this is the case, they do not extend this representation to equality of experience with their non-ADHD-affected peers, as evidenced by their acknowledgement of comparative deficits. Sciberras et al. (2011) reported that in their sample, self-worth was higher in children who reported higher QoL scores than their parents, compared with children who rated their QoL as worse than their parents, which may account for some of their apparent resilience.

Children’s self-reports may also be biased by their ADHD symptomatology. Children with this diagnosis are typically impulsive and have attentional difficulties, which may cause them to record responses in haste with little deliberation. In this sense, ADHD may limit their capacity to reflect on the ‘bigger picture’ of their life experiences, instead answering questions based on their immediate feelings. Thaulow and Jozefiak (2012) theorise that children with ADHD are more likely to focus on aspects of the present moment, while their parents are likely to focus on the child’s future, concerned by problems related to school and peer relationships. However, in this case, one might in fact expect a reversed trend to the one observed. That is, immediate events may make it more likely that children will rate their QoL less positively than their parents.

Parental perceptions are also open to bias. Some researchers have noticed a higher presence of psychopathology in parents of children with ADHD (Barkley et al. 1990; Biederman 1992). Parents of children with ADHD also experience more parenting stress than parents of healthy controls, similar to parents of other clinically referred children (Theule et al. 2010). Children with ADHD may have overly optimistic views. However, the views of their highly stressed parents may be influenced by negative thinking patterns that often underlie highly prevalent psychological problems. This hypothesis would fit with studies that have reported a link between parental emotional distress and more negative perceptions of their child’s QoL for other conditions (Janicke et al. 2007; Kobayashi and Kamibeppu 2011). Klassen et al. (2006) reported that when a psychosocial stressor was present, children rated their behaviour higher and their physical function lower compared with their parents’ ratings. It is likely that both stressful life events and parental mental health issues could inhibit communication between parents and children and thus affect the degree to which they are attuned. Further, parents who are already emotionally burdened may experience more distress related to their child’s ADHD behaviours and therefore perceive them as more severe and disruptive than the child experiences them to be.

### Agreement on specific domains

Just over one-third of the studies which reported domain scores found greater parent–child agreement on physical health domains as opposed to psychosocial domains (e.g. social, emotional and school experience). However, this trend should be interpreted cautiously as there were also studies where discrepancies were present across all domains or no domains. Authors of related reviews have suggested that the level of agreement on specific domains may depend on their clinical relevance to a particular disease group (Upton et al. 2008; Varni et al. 2003). They suggest that agreement is likely to be stronger on relevant domains because parents would be more involved in this aspect of the child’s health care. If this were the case, one might predict that in the case of ADHD, there would be greater agreement between parent and child ratings of child QoL for psychosocial rather than physical domains, since physical health is relatively unchanged by ADHD symptomatology. However, this review has found no evidence to support this theory in the context of ADHD. It may be that psychosocial domains which incorporate emotional, social and school experiences are more subjective and therefore open to parental interpretation, while physical health is easier for parents to objectively assess.

### Agreement and co-morbidities

It is difficult to make inferences regarding the impact of co-morbidities on parent–child agreement as few studies reported direct comparisons between ADHD only groups and co-morbid groups. Half of the studies which used ADHD only samples reported significant discrepancies between scores, while agreement across samples where co-morbidities had not been excluded was also variable, and samples were not homogenous in this regard. However, two potentially important findings with regard to co-morbidities were highlighted in the review. Klassen et al. (2006) found that children with co-morbid ODD/CD rate their mental health and behaviour more highly than their parents. It would thus be easy to imagine that the additional stress of co-morbidities further reduces communication and therefore agreement between parent and child. However, Thaulow and Jozefiak (2012) found that children with ADHD without co-morbidities self-rated their QoL higher than children with anxiety or depression, while there was no difference between these groups according to parent-reported ratings. This latter finding gives additional support to the theory that children with ADHD, unencumbered by co-morbid psychiatric problems, have a more positive outlook on their lives than their parents expect. In contrast, children with emotional problems such as anxiety or depression are more likely to view their lives more negatively and more in line with their parents’ expectations.

The two findings appear at first to sound contradictory, as surely if optimism is highest when ADHD is ‘pure’, parent–child agreement would be predicted to improve as co-morbidity increases. Yet the symptoms of ODD and CD are also externalising, and rather than affecting the coping style of the young person (as an internalising emotional disorder might), they may simply be adding to the stress of the parent and/or serving to reinforce the positive illusory coping mechanism within the child, creating further discrepancy. Perhaps then, the nature of ADHD symptomatology, e.g. externalising symptoms (hyperactivity) versus internalising symptoms (negative cognitions) could, relative to other mental health problems, be a protective factor for a child’s perception of their QoL. Reservedly, this hypothesis is based on the findings of only two studies. More research is needed to examine the impact of co-morbidities on parent–child agreement levels. In particular, studies that utilise comparative data across different conditions and that consider their impact on both parents and children, are of interest.

### Agreement across QoL measures

Over two-thirds of the included studies used the Peds-QL as the QoL measure. A potential explanatory factor for some of the preference for this measure is that the author of the Peds-QL is also an author on three of the included studies. A clear benefit of having such a high proportion of studies utilise the same measure, was that it allowed comparisons to be made both within and between QoL measures. Upton et al. (2008) suggested that the Peds-QL has a relatively high number of items which measure observable behaviours and that this may result in greater agreement between parents and children on this measure. The findings of this review contradict this premise, evidenced in the fact that two-thirds of the Peds-QL studies reported significant disagreement in overall QoL ratings of parents and children. Both of the ILC studies and the CHQ study reported poor concordance between raters, and the TACQOL and DUX-25 reported discrepancies on a number of domains. Therefore, it appears that the trend of discrepancies observed across studies cannot readily be attributed to the QoL measure specified.

Of interest, in Danckaerts et al.’s (2010) review, in the two studies which utilised the CHQ, children did not rate their QoL differently from controls, while the four others (which used other QoL measures) reported reduced QoL. In the current review, a similar pattern was observed. Only one study utilised the CHQ, and it was the only one (of those who reported comparisons with normative data) which did not observe reduced QoL in children with ADHD. The eight studies which reported impaired QoL utilised other QoL measures (Peds-QL, ILC, TACQOL and DUX-25). However, the CHQ study utilised population norms from a different country, meaning issues such as dissimilar healthcare systems and socio-economic status could result in key differences between the QoL of the children in the samples. However, it should also be made explicit that the (Klassen et al. 2006) study was reviewed as part of both Danckaerts et al.’s (2010) review and the current review; therefore, more comparisons featuring studies which utilise CHQ self-report measures are necessary before conclusions can be drawn.

### Strengths and limitations of the review

The search strategy utilised was comprehensive, and studies were screened and included from a variety of sources. Additionally, a second rater independently appraised the conformity of a proportion of the included studies, and inter-rater reliability checks were performed, limiting appraisal bias. However, the authors acknowledge that only one individual was responsible for selecting studies based on inclusion and exclusion criteria and that ideally this would be cross-checked. All of the included studies utilised standardised QoL instruments with established psychometric properties, thus refining the validity and reliability of the available data.

A limitation of all survey-based research is responder bias and the lack of available comparison data regarding why some and not others partake in the research. Inconsistencies between parent and child ratings may reflect sample differences. Samples had variable inclusion and exclusion criteria, age and gender distributions, and response rates. The reviewed research studies include samples which are internationally diverse, and participants are often treated within dissimilar healthcare systems. Diagnostic inconsistencies including the use of ICD-10 or DSM-IV criteria, the level of clinician experience and the use of research-specific criteria in some cases, will inevitably have led to some incongruence between samples. The authors acknowledge that it would have been useful to include a section in the quality criteria relating to how ADHD diagnosis was assigned in each sample. Further, diagnostic criteria have changed over time, and the search terms may have missed studies that utilised previous terminology for ADHD.

Participants were generally recruited by convenience sampling methods with little randomisation. In addition, some samples will have a referral bias for more complex/co-morbid cases depending on the recruitment method, the stage of their treatment and when they received a diagnosis. Some children completed questionnaires unaided or online, while researchers provided assistance to others or utilised an interview format. The method was usually based on the age of the child. Given the attentional problems associated with this population, the method of completion may have impacted on the child’s QoL ratings, with children potentially being inhibited by the presence of a researcher or by improving their attention. However, the directional impact and magnitude of each of these scenarios on the child’s QoL ratings are unknown. Notably, due the high proportions of boys within the samples, findings may not be generalisable to girls with ADHD. In addition, due to lack of information and comparison, the authors are unable to comment on the differences between child and adolescent data in comparison with parental reports. This would be of interest given that parents are likely to have less awareness of the QoL of adolescents than children.

### Implications for clinical practice

In addition to their application in research, QoL measures can be of value to clinicians working with families with a child with ADHD. They might highlight specific areas where a child is having difficulty and thus where appropriate support can be sought out and targeted. Although ADHD symptoms are often reduced by medication and other psychosocial treatment interventions, it is equally important to investigate and consider areas of a child’s life where there may be associated distress that might be reduced. Further, given the apparent discrepancies between parent and child perceptions of the QoL of children with ADHD found in published research, it may be helpful for clinicians to explore these differences on an individual level. Such investigations may illicit a clearer understanding of the impact of ADHD on the perceptions of the individual members of the family. If the child indicates that they experience life more positively than parents predict, this may in itself alleviate some distress in parents. It may also allow clinicians and parents to challenge or modify their own expectations in the light of the child’s own views.

Parents will vary in terms of their sensitivity and understanding of their child’s subjective well-being. However, substantial discrepancies across a range of domains could signpost relational issues between a parent and child that could be further examined and potentially addressed. We recommend that dual informants are always utilised when possible and that measures are interpreted with caution, given the potential sources of reporting bias on both parts. Further, given that the child’s accessing of services is usually predicted by parental concerns regarding the child’s QoL, it may be helpful for clinicians to reflect that there is perhaps no ‘true’ depiction of the child’s QoL, rather than both views should be valued and validated as integral contributions to clinical assessment and treatment planning.

### Implications for future research

Studies and reviews comparing parent/child agreement across different health conditions have mostly considered children with physical health conditions. Further studies which directly compare agreement between parents and children on QoL measures across samples of children with a range of psychiatric diagnoses may aid understanding of the potential impact of each set of symptoms. For example, if levels of agreement between parents and children vary between samples of children with depression (internalising symptoms), conduct disorders (externalising symptoms) and OCD (internalising and externalising symptoms), we could learn a great deal about how children’s perceptions (relative to their parents) are impacted by their condition and perhaps learn more about how each condition affects the parent/child relationship. Further attention should be given to the potential sources of bias for both informants. Large quantitative studies investigating the specific impact of parental stress on parent and child ratings of child QoL would be of interest.

Previous research found little differences between mothers and fathers’ ratings of QoL in population samples (Jozefiak et al. 2008). However, this trend may be different when a child has a health condition given that one parent may be more involved with the child’s health care. Therefore, studies which compare proxy-raters in terms of their relationship with the child may be of interest, along with studies which explore agreement associated with child gender and age. Given the highly co-morbid nature of ADHD, more studies directly comparing agreement between ADHD only samples and samples according to type and number of co-morbidities may also be of value. Since symptom severity is generally rated by parents in research (Danckaerts et al. 2010), such ratings may be open to the same potential sources of bias QoL ratings and may result in erroneous correlations between ADHD symptoms and QoL. Teacher- or clinician-based ratings would be preferable if investigating the impact of symptom severity on agreement levels. Finally, qualitative studies considering the basis on which both sets of informants assess the child’s QoL would be highly advantageous in helping to establish the cognitive processes behind parent and child perceptions.

## Conclusions

Previous related reviews have focussed on agreement across multiple diagnoses (where only one ADHD study was included) (Eiser and Morse 2001) or have utilised mainly proxy-reports when describing child QoL (Danckaerts et al. 2010). Thus, it had formerly been difficult to establish a clear picture of children’s views of their QoL, both in relation to their non-ADHD affected peers and to their parents. This review adds to the current evidence base by bringing together the existing published research *specific* to the QoL of children with a diagnosis of ADHD and by representing and comparing the views of both parents *and* children. In summary, this review found that there is consistent uni-directional evidence that children with ADHD perceive their QoL more favourably than their parents do, but less favourably than healthy controls. Thus, parent and child ratings of QoL should not be considered interchangeable when assessing the QoL of children with ADHD. Rather both should be considered as unique and valuable perspectives for clinical and research purposes.

## Appendix: STROBE statement—checklist of items that should be included in reports of observational studies

**Table:**

	Item no.	Recommendation
Title and abstract	1	(a) Indicate the study’s design with a commonly used term in the title or the abstract (b) Provide in the abstract an informative and balanced summary of what was done and what was found
Introduction		
Background/rationale	2	Explain the scientific background and rationale for the investigation being reported
Objectives	3	State-specific objectives, including any prespecified hypotheses
Methods		
Study design	4	Present key elements of study design early in the paper
Setting	5	Describe the setting, locations and relevant dates, including periods of recruitment, exposure, follow-up and data collection
Participants	6	(a) *Cohort study*—Give the eligibility criteria, and the sources and methods of selection of participants. Describe methods of follow-up *Case*–*control study*—Give the eligibility criteria, and the sources and methods of case ascertainment and control selection. Give the rationale for the choice of cases and controls *Cross*-*sectional study*—Give the eligibility criteria, and the sources and methods of selection of participants (b) *Cohort study*—For matched studies, give matching criteria and number of exposed and unexposed *Case*–*control study*—For matched studies, give matching criteria and the number of controls per case
Variables	7	Clearly define all outcomes, exposures, predictors, potential confounders and effect modifiers. Give diagnostic criteria, if applicable
Data sources/measurement	8^a^	For each variable of interest, give sources of data and details of methods of assessment (measurement). Describe comparability of assessment methods if there is more than one group
Bias	9	Describe any efforts to address potential sources of bias
Study size	10	Explain how the study size was arrived at
Quantitative variables	11	Explain how quantitative variables were handled in the analyses. If applicable, describe which groupings were chosen and why
Statistical methods	12	(*a*) Describe all statistical methods, including those used to control for confounding (*b*) Describe any methods used to examine subgroups and interactions (*c*) Explain how missing data were addressed (*d*) *Cohort study*—If applicable, explain how loss to follow-up was addressed *Case*–*control study*—If applicable, explain how matching of cases and controls was addressed *Cross*-*sectional study*—If applicable, describe analytical methods taking account of sampling strategy (e) Describe any sensitivity analyses
Results		
Participants	13^a^	(a) Report numbers of individuals at each stage of study—e.g. numbers potentially eligible, examined for eligibility, confirmed eligible, included in the study, completing follow-up, and analysed (b) Give reasons for non-participation at each stage (c) Consider use of a flow diagram
Descriptive data	14^a^	(a) Give characteristics of study participants (e.g. demographic, clinical, social) and information on exposures and potential confounders (b) Indicate number of participants with missing data for each variable of interest (c) *Cohort study*—Summarise follow-up time (e.g. average and total amount)
Outcome data	15^a^	*Cohort study*—Report numbers of outcome events or summary measures over time *Case*–*control study*—Report numbers in each exposure category or summary measures of exposure *Cross*-*sectional study*—Report numbers of outcome events or summary measures
Main results	16	(a) Give unadjusted estimates and, if applicable, confounder-adjusted estimates and their precision (e.g. 95% confidence interval). Make clear which confounders were adjusted for and why they were included (b) Report category boundaries when continuous variables were categorised (*c*) If relevant, consider translating estimates of relative risk into absolute risk for a meaningful time period
Other analyses	17	Report other analyses done—e.g. analyses of subgroups and interactions, and sensitivity analyses
Discussion		
Key results	18	Summarise key results with reference to study objectives
Limitations	19	Discuss limitations of the study, taking into account sources of potential bias or imprecision. Discuss both direction and magnitude of any potential bias
Interpretation	20	Give a cautious overall interpretation of results considering objectives, limitations, multiplicity of analyses, results from similar studies and other relevant evidence
Generalisability	21	Discuss the generalisability (external validity) of the study results
Other information		
Funding	22	Give the source of funding and the role of the funders for the present study and, if applicable, for the original study on which the present article is based

An explanation and elaboration article discusses each checklist item and gives methodological background and published examples of transparent reporting. The STROBE checklist is best used in conjunction with this article (freely available on the websites of PLoS Medicine at http://www.plosmedicine.org/, Annals of Internal Medicine at http://www.annals.org/ and Epidemiology at http://www.epidem.com/). Information on the STROBE Initiative is available at www.strobe-statement.org

^a^Give information separately for cases and controls in case–control studies and, if applicable, for exposed and unexposed groups in cohort and cross-sectional studies
